# Soluble epoxide hydrolase inhibition preserves alveolar bone in experimental periodontitis with estrogen deficiency

**DOI:** 10.3389/fimmu.2025.1708504

**Published:** 2025-12-05

**Authors:** Charles Ritter, Pedro Silva Pacheco, Lila Batista Trajano Mattos, Mariana Quirino Silveira Soares, Debora Duarte Moreira, Izabel Regina Fischer Rubira-Bullen, Marco Antonio Hungaro Duarte, Jeroen Van Dessel, Bruce D. Hammock, Laura Carbone, Juliana Trindade Clemente-Napimoga, Henrique Ballassini Abdalla, Marcelo Henrique Napimoga

**Affiliations:** 1 Faculdade São Leopoldo Mandic, Campinas, Brazil; 2 Department of Surgery, Stomatology, Pathology, and Radiology, Bauru School of Dentistry, University of São Paulo, Bauru, São Paulo, Brazil; 3 Department of Dentistry, Endodontics and Dental Materials, Bauru School of Dentistry, University of São Paulo, Bauru, São Paulo, Brazil; 4 Department of Oral and Maxillofacial Surgery, University Hospitals Leuven, Leuven, Belgium; 5 OMFS-IMPATH Research Group, Department of Imaging and Pathology, Faculty of Medicine, KU Leuven, Leuven, Belgium; 6 Department of Entomology and Nematology and UCD Comprehensive Cancer Center, University of California, Davis, Davis, CA, United States; 7 Division of Rheumatology, Department of Medicine, Distinguished University Chair in Rheumatology, Medical College of Georgia at Augusta University, Augusta, GA, United States

**Keywords:** inflammation, osteoporosis -, periodontitis (inflammatory), soluble epoxide hydrolase (sEH) inhibitors, bone

## Abstract

Osteoporosis and periodontitis are highly prevalent chronic conditions characterized by deregulated bone remodeling. Estrogen deficiency after menopause accelerates systemic bone resorption and also increases susceptibility to periodontal breakdown, resulting in a clinically relevant comorbidity that amplifies alveolar bone loss. Therapeutic strategies that target both systemic and local inflammatory mechanisms remain scarce. Soluble epoxide hydrolase (sEH) regulates the degradation of epoxyeicosatrienoic acids, lipid mediators with anti-inflammatory properties. Its inhibition stabilizes these mediators and has emerged as a promising approach in chronic inflammatory diseases. Here, we investigated whether pharmacological sEH inhibition could attenuate periodontitis exacerbated by estrogen deficiency. Female Wistar rats (8 weeks, 250 g) were assigned to Sham, OVX, PD, OVX + PD, or OVX + PD treated with the sEH inhibitor TPPU (1 mg/kg, oral). Experimental periodontitis was induced by ligature placement around the first lower molar and evaluated at 14 and 28 days for alveolar bone loss. Histological analyses were performed on mandibles (H&E and immunohistochemistry). Gingival biopsies and cervical lymph node were used for gene expression and protein level. Here, we demonstrated that estrogen deficiency aggravated ligature-induced periodontal destruction, as evidenced by greater furcation area, increased osteoclast numbers, elevated pro-inflammatory cytokines, and a higher RANKL/OPG ratio, alongside suppression of osteogenic markers. TPPU significantly reversed these changes by reducing bone loss, downregulating inflammatory cytokines, normalizing RANKL/OPG balance, and enhancing osteoblast-related gene expression. Furthermore, TPPU decreased immune activation in draining lymph nodes, indicating systemic effects. In conclusion, sEH inhibition by TPPU attenuates estrogen deficiency–associated periodontitis, representing a potential therapeutic strategy for postmenopausal periodontal bone loss.

## 1 Introduction

Menopause represents a major physiological transition, generally occurring between 45 and 55 years of age, and is defined by the permanent cessation of menstruation due to depletion of ovarian follicular activity (1). The accompanying decline in estrogen and progesterone has systemic repercussions and also affects oral health, contributing to symptoms such as dry mouth, burning sensations, altered taste, and increased susceptibility to periodontal disease (2, 3).

Estrogen deficiency has a well-established impact on the periodontium. Reduced estrogen disrupts bone remodeling and accelerates bone mineral density loss, including in the alveolar bone, thereby compromising tooth support and increasing periodontitis risk (4). These hormonal changes also heighten the immune-inflammatory response to oral biofilms, promoting exaggerated periodontal inflammation and tissue breakdown (5). Thus, the interplay between estrogen decline, altered immunity, and alveolar bone resorption underscores the clinical relevance of investigating therapies capable of addressing both inflammation and bone loss in postmenopausal women.

Although antiresorptive medications remain central to osteoporosis management—such as bisphosphonates (alendronate, risedronate, ibandronate, zoledronic acid) and alternatives like denosumab or anabolic agents (teriparatide, abaloparatide, romosozumab)—their systemic effects and potential adverse events, including osteonecrosis of the jaw, complicate long-term use (6–8). Given that osteoporosis and periodontitis share mechanisms of dysregulated bone remodeling and inflammation (9), these therapies have also been evaluated for their influence on alveolar bone homeostasis (10). However, safer and more targeted strategies remain necessary.

In this context, inhibitors of soluble epoxide hydrolase (sEH) have emerged as promising candidates. By stabilizing epoxy fatty acids (EpFAs), including epoxyeicosatrienoic acids (EETs), sEH inhibition enhances inflammation resolution and modulates host responses (11, 12). We have previously demonstrated that sEH inhibitors attenuate alveolar bone loss by regulating immune pathways and increasing specialized pro-resolving mediators (11–15).

Therefore, this study evaluated the effects of sEH inhibition on ligature-induced periodontitis under estrogen-deficient conditions. Using an ovariectomized rat model that reproduces postmenopausal systemic bone loss and local periodontal inflammation, we investigated how sEH inhibition modulates endocrine–immune interactions and whether it represents a potential therapeutic approach to preserve alveolar bone in estrogen deficiency.

## 2 Materials and methods

### 2.1 Animals and ethical considerations

**Figure 1:**
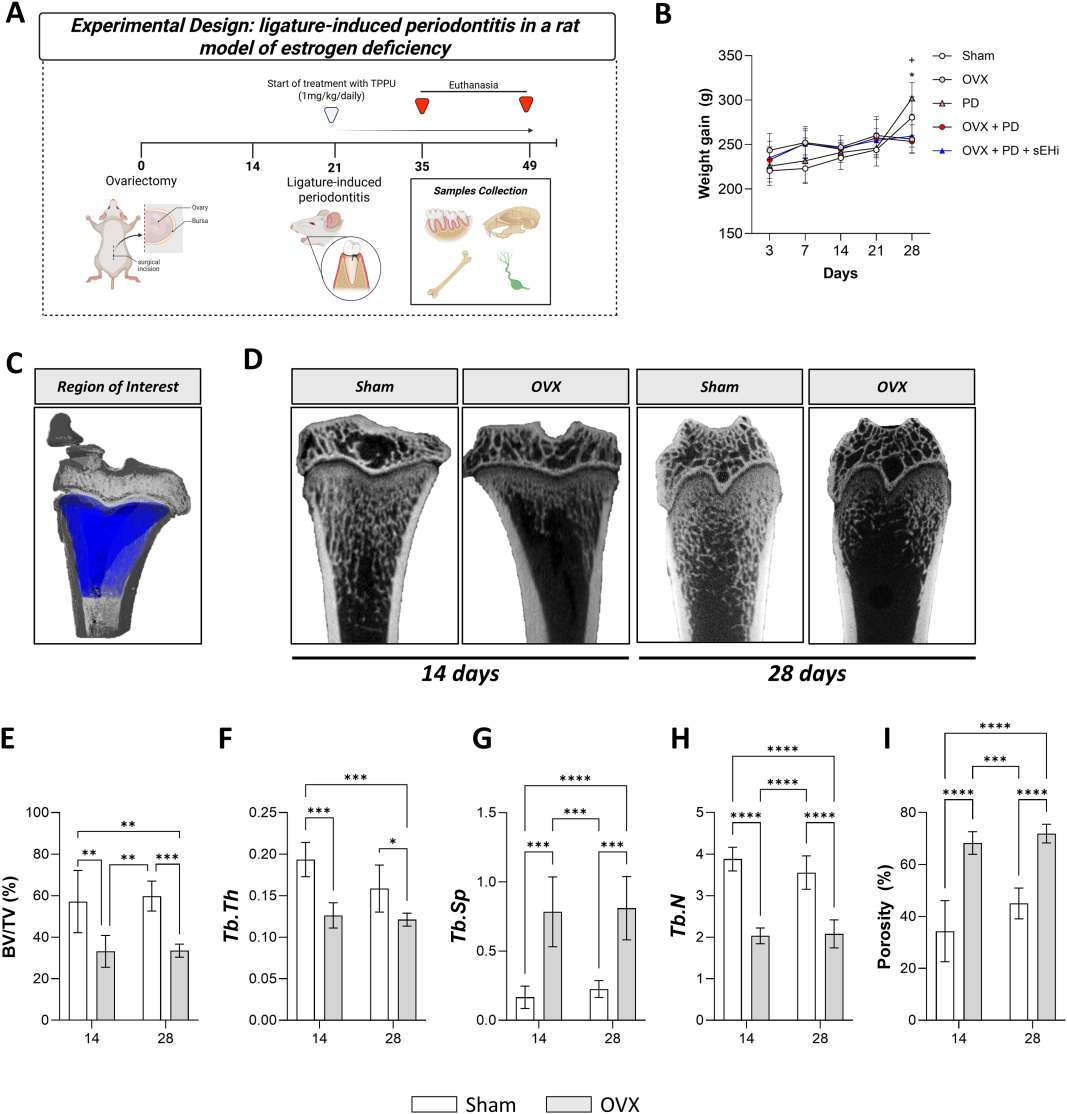
Experimental design and standardization of estrogen deficiency model. **(A)** Schematic representation of experimental design. **(B)** Animals weigh during experimental periodontitis protocol. **(C)** Representative image of the region of interest (ROI) for micro-computed tomography (µCT) analysis. **(D)** Three-dimensional reconstructions. **(E)** Bone density (BV/TV). **(F)** Trabecular thickness (Tb.Th). **(G)** Greater medullary spaces (Tb.SP). **(H)** Trabecular bone number. **(I)** Porosity (Tot.Po). Data are expressed as mean ± SD; n = 5 animals per group. *p<0.05, **p < 0.01, ***p < 0.001, ****p < 0.0001.

Female Wistar rats (Rattus norvegicus), aged 8 weeks and weighing approximately 250 g at baseline, were obtained from ANILAB, Paulínia – SP. Animals were housed in groups of 3–4 animals per cage under standard laboratory conditions, with controlled temperature (22 ± 2 °C), relative humidity (55 ± 10%), and a 12-h light/dark cycle. Food and water were provided *ad libitum*. All experimental procedures were performed in accordance with the ethical guidelines for animal experimentation and approved by the Institutional Committee for Animal Care and Use (2023/16). Care was taken to minimize animal discomfort, and all surgical interventions were performed under general anesthesia with appropriate post-operative monitoring. The animals were weighed throughout the experimental period (**Figure 1B**).

### 2.2 Acquisition and administration of the soluble epoxide hydrolase inhibitor (sEHi or TPPU)

The soluble epoxide hydrolase inhibitor 1-trifluoromethoxyphenyl-3-(1-propionylpiperidin-4-yl) urea (TPPU) was generously provided by Dr. Bruce Hammock (UC-Davis, CA – USA). The compound was administered at a dose of 1 mg/kg/day, as previously established by our group (11). The treatment regimen was initiated 2 hours prior to the induction of experimental periodontitis, in accordance with previous studies (11, 12, 16).

### 2.3 Experimental design

Rats were randomly assigned into five experimental groups: Sham-operated (Sham), ovariectomized without further intervention (OVX), experimental periodontitis disease alone (PD), ovariectomy combined with experimental periodontitis (OVX + PD), and ovariectomy plus periodontitis treated with sEHi (OVX + PD + sEHi). Two months after either bilateral ovariectomy or sham surgery, experimental periodontal disease was induced by ligature around the first lower molar. Animals were monitored and weight to secure general health and well-being throughout the experimental period. Subsets of animals were euthanized at two distinct time points—14 and 28 days following the induction of periodontal disease, in order to assess both early and late responses (17–19). Randomization was carried out using https://www.graphpad.com/quickcalcs/randomize1/. In addition, the animal’s procedures/treatments were performed by an investigator not involved in the experimental readouts, and all analyses were conducted in a blinded fashion. The experimental flowchart is shown in **Figure 1A**.

### 2.4 Ovariectomy procedure

Animals were anesthetized by intraperitoneal administration of ketamine (75 mg/kg; Dopalen^®^, Ceva, Brazil) combined with xylazine (10 mg/kg; Rompun^®^, Bayer, Brazil). Bilateral ovariectomy was performed through a dorsal approach in animals assigned to the ovariectomized groups. Control animals underwent sham surgery, which consisted of exposure of the ovaries followed by repositioning of the intact organs to their normal anatomical location. Postoperatively, animals received a single intramuscular dose of analgesic (0.05 mL/250 g body weight; Tramal^®^, Pfizer LTDA, New York, NY, USA) and antibiotic (0.25 mL/250 g body weight; Pentabiotico Veterinário, Zoetis LTDA, Parsippany, NJ, USA). The estrous cycle was monitored for two consecutive months following ovariectomy or sham surgery in order to confirm the success of the procedure and the estrogen-depleted status, as previously described by Marcondes and collaborators (20). Ovariectomized animals consistently exhibited a diestrus vaginal smear pattern, characterized by a predominance of polymorphonuclear leukocytes and low cellularity. In contrast, control animals displayed the four regular stages of the estrous cycle (estrus, diestrus, proestrus, and metestrus). In the present study, no animals were excluded due to unsuccessful ovariectomy.

### 2.5 Induction of experimental periodontitis

Animals were anesthetized with a single intramuscular injection of ketamine (75 mg/kg; Dopalen^®^, Ceva, Brazil) combined with xylazine (10 mg/kg; Rompun^®^, Bayer, Brazil). Experimental periodontitis was induced using the ligature model, which promotes endogenous microbial dysbiosis rather than relying on exogenous inoculation of bacteria loading (21). For this, a sterile cotton ligature was carefully placed in a subgingival position, encircling the first lower molar. It is essential to mention that the knots were tied enough to hold in the position and floppy enough not to induce gingival ischemia.

### 2.6 Sample collection and processing

At the end of the experimental periods (14 or 28 days after ligature placement), animals were euthanized by deepening of anesthesia in an isoflurane-saturated chamber (2%), followed by cervical dislocation. Femur, maxillae, gingival tissues, and cervical lymph nodes were collected. Tissue samples intended for gene expression analysis were immediately stored at −80 °C. Samples designated for histological sections and micro–computed tomography (micro-CT) analysis was fixed in 10% neutral buffered formalin.

### 2.7 Micro–computed tomography

Samples were scanned using a SkyScan 1174 micro-computed tomography system (Bruker, Belgium) at 50 kV and 800 µA, with a 306° rotation and an angular step of 0.5°. Images were acquired with an isotropic voxel size of 19.7 µm. A 0.5-mm aluminum filter was applied to minimize beam-hardening artifacts. All images were reconstructed using the NRecon software (Bruker, Belgium), and defects were manually registered with the DataViewer software (Bruker, Belgium). A standardized region of interest (ROI) was defined to encompass the entire 5-mm defect diameter and the full thickness of the alveolar bone at the defect site. This ROI was individually adjusted for each sample. Image segmentation was performed by a single blinded evaluator using a semi-automatic thresholding algorithm in CTAnalyser software (Bruker, Belgium). Morphometric parameters were then calculated, including bone volume fraction (BV/TV, %), total porosity (Po[tot], %), bone surface density (BS/TV, %), trabecular thickness (Tb.Th, mm), trabecular number (Tb.N, 1/mm), trabecular separation (Tb.Sp, mm), and connectivity density (Conn.Dn, 1/mm³).

### 2.8 Bone loss measurement by methylene blue

At the end of the experimental period, animals were euthanized, and the mandibles were carefully removed. To enable bone loss evaluation, the specimens were immersed overnight in 3% hydrogen peroxide, followed by mechanical dissection to remove residual soft tissues. Mandibles were subsequently stained with 0.5% methylene blue, allowing clear visualization of the cementoenamel junction. The lingual surfaces were captured using a digital camera (Canon EOS 1000D) coupled to a stereomicroscope (ZEISS CL 1500 ECO) at 2.5× magnification. Bone loss was quantified using ImageJ software by measuring the distomesial area (mm²) between the cementoenamel junction and the alveolar bone crest on the lingual aspect of the first molar (21, 22).

### 2.9 Histological analysis

Following deep anesthesia, animals were euthanized and the mandibles were carefully dissected. Specimens were fixed in 10% neutral buffered formalin for 48 hours, rinsed in running water, dehydrated through graded alcohols, and embedded in paraffin. Serial sagittal sections of 6 μm thickness were obtained, mounted on glass slides, and stained with hematoxylin and eosin (H&E) for histological evaluation of bone resorption. For the histomorphometric assessment of bone loss in the furcation region, three linear measurements were obtained: the first adjacent to the mesial root, the second at the center of the furcation, and the third adjacent to the distal root. The mean value of these three measurements was calculated and considered as the extent of bone loss, expressed in millimeters (mm). Histometric assessment was performed using ImageJ software. In addition, H&E-stained sections were examined to investigate the formation of multinucleated cells. Osteoclast-like cells—defined as multinucleated cells containing three or more nuclei—were identified and counted in the alveolar bone adjacent to the tooth roots at 200× magnification.

For the immunohistochemistry, mandibles were fixed with 10% formaldehyde, and then conventionally dehydrated and paraffin embedded. Afterward, the sections were deparaffinized and hydrated, and 3 immersions of the slides quenched endogenous peroxidase activity in 3% hydrogen peroxide for 5 min each. The blocking was made with 5% non-fat dry milk for 30 min. The antigen retrieval was made in a steamer with citrate buffer (pH 6.0) for 20 min at 97°C and 10 min at room temperature. The mandibles sections were then incubated overnight at 4°C with the following primary antibodies diluted in EnVision FLEX Antibody Diluent (DaKo, K8006): RANKL (1:50, Sc-7828, Santa Cruz Biotechnology), and OPG (1:300; GTX 55734; Genetex). After primary antibodies incubation, the sections were then incubated with secondary antibody Advance HRP Detection System (Dako Corp., Carpinteria, CA, USA), treated with DAB (3,30-diam-inobenzidine tetrahydrochloride Dako Corp., Carpinteria, CA, USA) for 10 min and counter-stained with Mayer’s Hematoxylin for 5 min at room temperature. Images from the region of interest were obtained using an optical microscope using a Zeiss Axioskop 2 Plus microscopy (Zeiss, GmbH, Germany).

### 2.10 Reverse transcriptase-polymerase chain reaction

For the *in vivo* data, gingival tissue and cervical lymph nodes were collected and processed for RNA extraction using the Illustra RNAspin Mini Kit (GE Healthcare, Milwaukee, WI, USA), following the manufacturer’s instructions. Total RNA quantification was carried out using a spectrophotometer (GE Healthcare) at different wavelengths (260, 280, 230, and 320 nm). Subsequently, cDNA synthesis was performed using 1 μg of total RNA in the Veriti^®^ Thermal Cycler (Applied Biosystems) with the reverse transcriptase enzyme from the RevertAid First Strand cDNA Synthesis Kit (Thermo Fisher Scientific), yielding a final concentration of 40 ng of cDNA. Relative quantification of the amplified product was performed using GAPDH as the reference gene, and gene expression analysis was conducted using the 2-ΔΔCt method.

### 2.11 Cytokines quantification

Gingival tissue and cervical lymph nodes were collected according to protocols previously established (11, 23). Following euthanasia, marginal and attached gingiva surrounding the tooth of interest were carefully dissected using a #15C scalpel blade, ensuring removal of all soft tissue directly adherent to the dental and alveolar surfaces while avoiding contamination from adjacent mucosa. Deep cervical lymph nodes draining the periodontal region were exposed by blunt dissection in the submandibular and upper cervical areas and isolated free of surrounding connective and adipose tissue. Each sample (gingiva or lymph node) was immediately placed on ice and individually homogenized in 300 μL of RIPA buffer contained protease inhibitor (1:1000) until complete lysis. Homogenates were centrifuged at 10,000 rpm for 10 min at 4 °C, and supernatants were collected and stored at –20 °C until protein analysis. Levels of TNF-α, IL-1β, IL-6, IL-17 and TGFβ1 were quantified using commercial ELISA kits following the manufacturer’s instructions, and results were expressed as pg/mL.

### 2.12 Statistical analysis

The statistical power analysis was estimated using the software BioEstat 5.0. The ANOVA sample size test (analysis of variance) with a significance level of α = 0.05 and power of 80% was used based on a previous publication (17). The minimum difference between treatment means was = 0.15, while the standard deviation was set at 0.22. A minimum of 5 animals per group was required. Data were evaluated using one-way analysis of variance (ANOVA). Multiple comparisons were made using Tukey’s test. Student’s t-test were used when no more than 2 groups were compared. The significance level was set at p <.05 for all tests. Data were presented as mean ± standard deviation. GraphPad Prism 10.4.1 software was used for statistical calculations and graph plotting.

## 3 Results

### 3.1 Ovariectomy exacerbates periodontal bone resorption, whereas soluble epoxide hydrolase inhibition restores bone homeostasis

Initially, to ensure that ovariectomy successfully induced estrogen deficiency and thereby reproduced an osteoporotic condition in animals, femurs from Sham and OVX groups were collected and analyzed by micro-computed tomography (micro-CT) to evaluate bone architecture. The region of interest (ROI) was set at the distal femoral epiphysis (**Figure 1C**). Representative images were shown in **Figure 1D**. On 14 days, OVX rats displayed reduced BV/TV compared with Sham (p<0.05) and on 28 days, OVX showed significantly lower BV/TV relative to Sham (p<0.05) (**Figure 1E**). Trabecular thickness (Tb.Th) decreased in OVX animals at 14 and 28 days compared with controls (p<0.05) (**Figure 1F**). On the other hand, trabecular separation (Tb.Sp) was elevated in OVX animals at 14 and 28 days compared to Sham (p<0.05) (**Figure 1G**). Moreover, trabecular number was reduced in OVX animals at 14 and 28 days compared to Sham (p<0.05) (**Figure 1H**); For last, bone porosity was consistently higher in OVX animals at both time points versus Sham (p<0.05) (**Figure 1I**).

**Figure 2:**
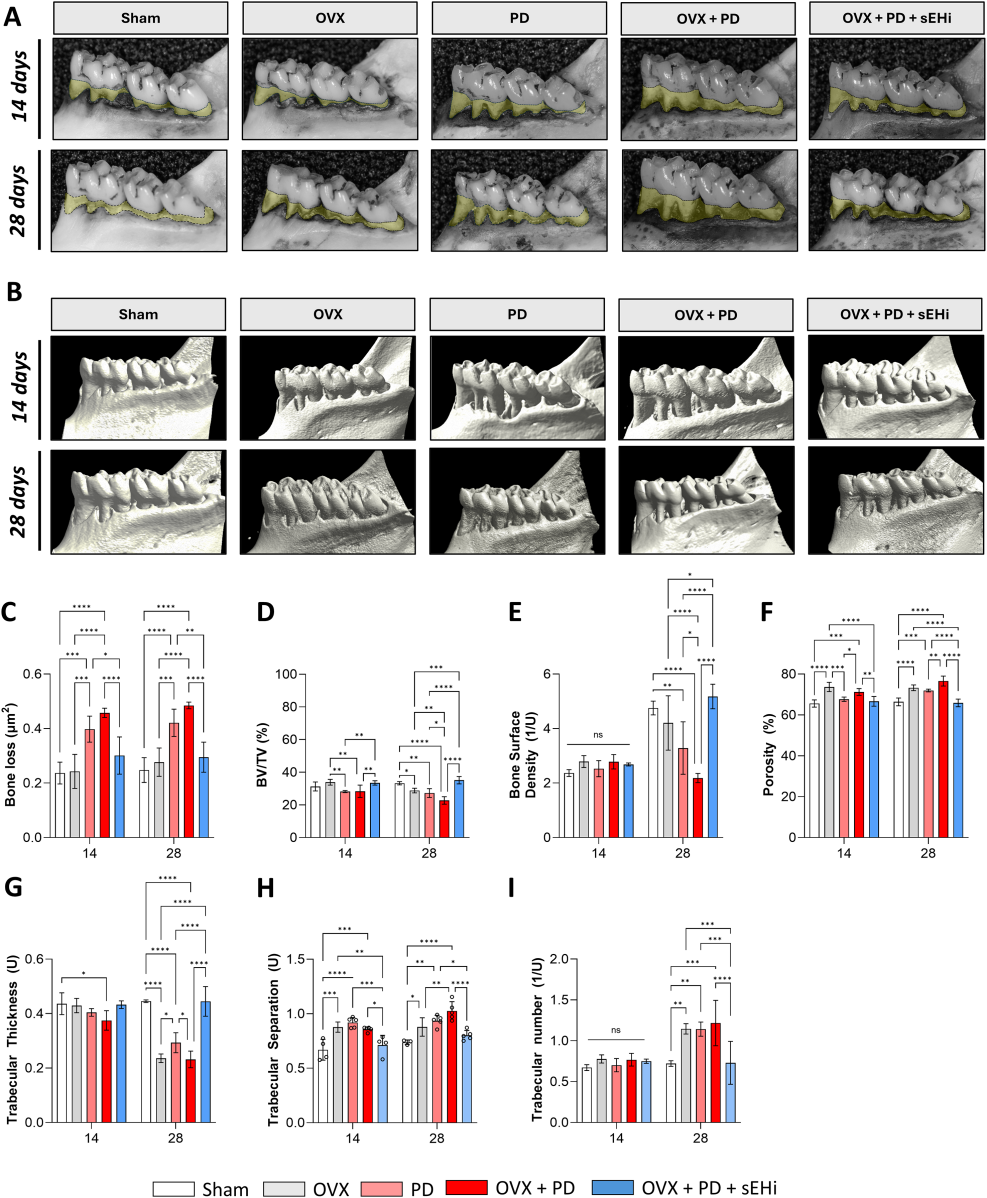
Targeting soluble epoxide hydrolase mitigates periodontal bone loss in ovariectomized rats with experimental periodontitis. **(A)** Bone loss was quantified as the area (mm2) between the cementum-enamel junction using methylene blue stain. Representative images from a buccal view of molars. The areas measured are highlighted in light yellow. **(B)** Micro-computed tomography (micro-CT) was used to quantify bone volume and representative micro-CT-3-dimensional reconstruction images of buccal molars was demonstrated. **(C)** Bone loss heigh measurement quantified using methylene blue stain. **(D)** Bone density (BV/TV). **(E)** Complexity of bone structure (BS/TV). **(F)** Porosity (Tot.Po). **(G)** Trabecular thickness (Tb.Th). **(H)** Greater medullary spaces (Tb.SP). **(I)** Trabecular bone number. Data are expressed as mean ± SD; n = 5 animals per group. *p<0.05, **p < 0.01, ***p < 0.001, ****p < 0.0001.

After confirming that ovariectomy induced significant alterations in bone architecture, we next investigated whether estrogen deficiency could further exacerbate experimental periodontitis–induced alveolar bone loss and assessed the contribution of sEH to this process. Mandibles were first stained with methylene blue for two-dimensional quantification of alveolar bone loss, with representative images shown in **Figure 2A**. Consistently across both experimental time points, PD and OVX+PD groups exhibited significantly greater alveolar bone loss compared with Sham and OVX animals (p<0.05), whereas pharmacological inhibition of sEH effectively reversed this loss (p<0.05) (**Figure 2C**), highlighting its protective role.

To further characterize the underlying microarchitectural changes, micro-CT analysis of affected mandibles was performed, and representative reconstructed images are presented in **Figure 2B**. At 14 days, BV/TV was significantly reduced in PD and PD+OVX animals compared with OVX alone p<0.05), and relative to PD+OVX+sEHi (p<0.05), indicating that the combination of periodontitis and estrogen deficiency potentiates early bone loss, which was attenuated by sEHi treatment (**Figure 2D**). By 28 days, OVX, PD, and PD+OVX animals exhibited decreased BV/TV compared with Sham, with PD+OVX showing further reduction relative to OVX alone (p<0.05) (**Figure 2D**); notably, these groups also displayed significantly lower values compared with PD+OVX+sEHi (p<0.05), underscoring the protective effect of the inhibitor (**Figure 2D**). Bone surface density followed a similar pattern, with no early differences but significant reductions at 28 days in PD and PD+OVX versus Sham (p<0.05), and in OVX, PD, and PD+OVX versus PD+OVX+sEHi (p<0.05) (**Figure 2E**). Porosity analyses further supported these findings: at 14 days, OVX and PD+OVX groups showed increased porosity compared with Sham (p<0.05), whereas PD exhibited reduced porosity compared with OVX but PD+OVX animals presented higher porosity than PD alone (p<0.05) (**Figure 2F**); importantly, OVX and PD+OVX remained significantly elevated compared with PD+OVX+sEHi. At 28 days, porosity levels in PD+OVX+sEHi were indistinguishable from Sham, while OVX, PD, and PD+OVX all exhibited sustained increases (p<0.05), reinforcing the long-term protective role of the inhibitor (**Figure 2F**). Trabecular thickness was reduced only in PD+OVX animals at 14 days (p<0.05), but by 28 days, OVX, PD, and PD+OVX all presented marked decreases compared with PD+OVX+sEHi (p<0.05), whereas the latter remained comparable to Sham (p<0.05) (**Figure 2G**). Trabecular separation was consistently increased in OVX, PD, and PD+OVX groups at both time points (p<0.05) compared with Sham and PD+OVX+sEHi, indicating a progressive deterioration of trabecular organization that was mitigated by treatment (p<0.05) (**Figure 2H**). Finally, trabecular number showed no changes at 14 days, but by 28 days OVX, PD, and PD+OVX displayed increased trabecular counts relative to Sham, while PD+OVX+sEHi animals remained similar to controls (p<0.05), suggesting that in the presence of sEH enzyme, disease conditions promote a maladaptive remodeling characterized by thinner and more numerous trabeculae (**Figure 2I**).

### 3.2 sEH inhibition reduces furcation bone loss, multinucleated cells, and inflammatory osteolytic factors

**Figure 3:**
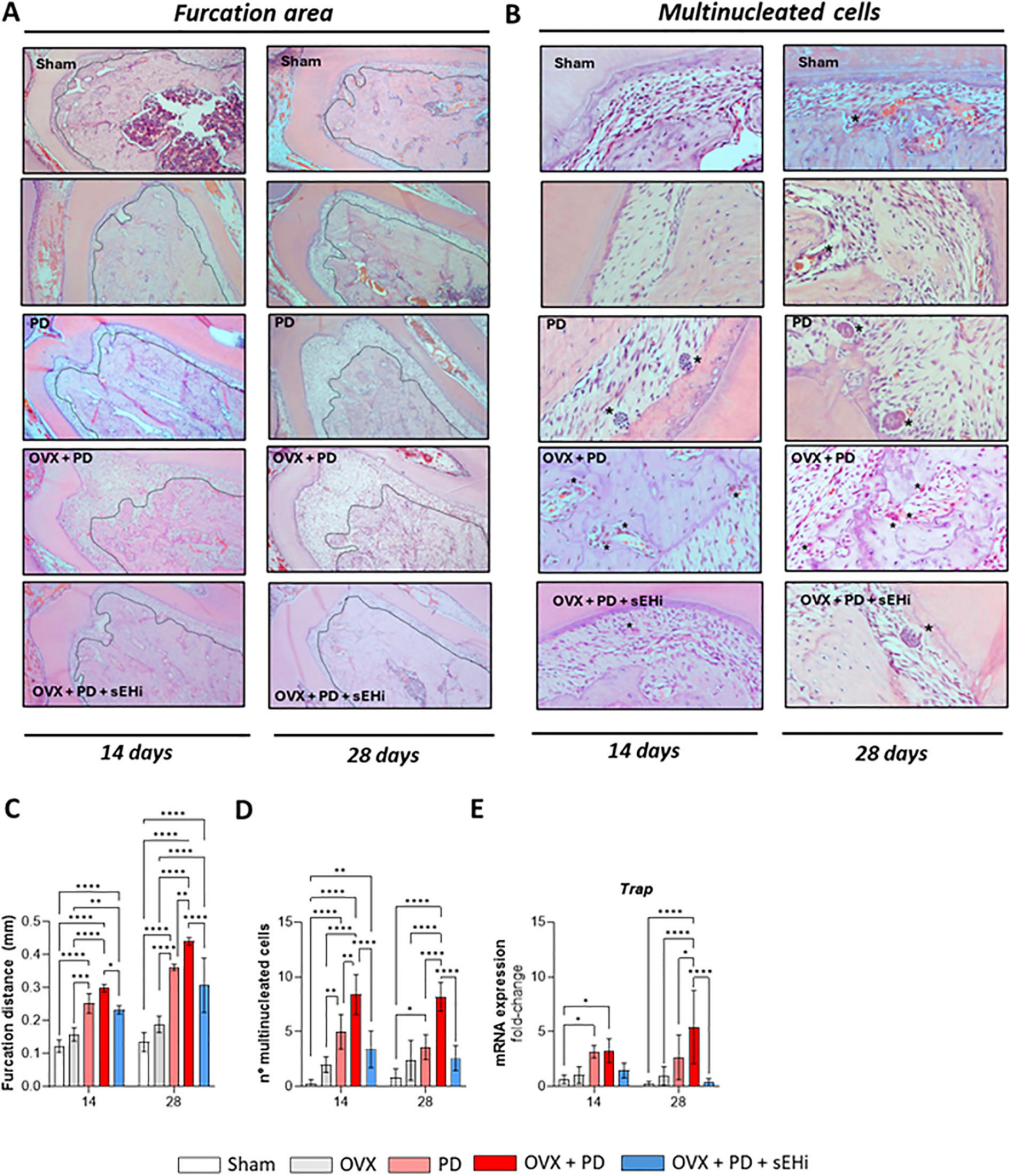
sEH inhibition prevents furcation destruction and multinucleated osteoclast formation in experimental periodontitis under estrogen deficiency. **(A)** Histological section of the furcation area around the first lower molar. Magnification: x40 **(B)** Representative histological sections indicating the presence or absence of multinucleated cells (asterisks). Magnification: x200. **(C)** Quantification of bone loss in the furcation region. **(D)** Quantification of multinucleated cells surrounding the first lower molar, in regions where ligatures were applied. Gene expression of Trap was measured **(E)**. Data are expressed as mean ± SD; n = 5 animals per group. *p<0.05, **p < 0.01, ***p < 0.001, ****p < 0.0001.

To further investigate the impact of estrogen deficiency on periodontal bone loss, we performed histological analysis of the furcation region—a site commonly affected in advanced periodontal disease—and quantified multinucleated osteoclast-like cells. Representative images are shown in **Figures 3A, B**. At 14 days, both PD and OVX+PD groups exhibited significantly greater bone loss compared with Sham and OVX, while pharmacological inhibition of sEH attenuated this effect (p<0.05; **Figure 3C**). The same pattern persisted at 28 days, with PD and OVX+PD showing marked bone loss that was significantly reduced by sEH inhibition (p<0.05; **Figure 3C**). Osteoclasts, multinucleated cells central to bone remodeling and pathological resorption (24), were quantified in parallel (**Figure 3D**). At 14 days, PD and OVX+PD groups showed a higher number of multinucleated cells than the other groups (p<0.05), with OVX+PD presenting significantly greater numbers than PD alone (p<0.05), supporting the notion that estrogen deficiency facilitates osteoclastogenesis and contributes to bone loss. At 28 days, this pattern remained, as PD and OVX+PD groups exhibited increased osteoclast-like cells (p<0.05), whereas sEH inhibition effectively reduced their numbers (p<0.05; **Figure 3D**). Furthermore, we examined the expression of key osteolytic markers in gingival tissue. Tartrate-resistant acid phosphatase (TRAP), an enzyme associated with osteoclast activity and number (25), was significantly upregulated in PD and OVX+PD groups at both experimental time points (p<0.05). Notably, sEH inhibition reduced Trap expression only at 28 days after periodontitis induction (p<0.05; **Figure 3E**). In parallel, we evaluated the RANKL/OPG signaling axis, a central pathway in bone remodeling.

**Figure 4:**
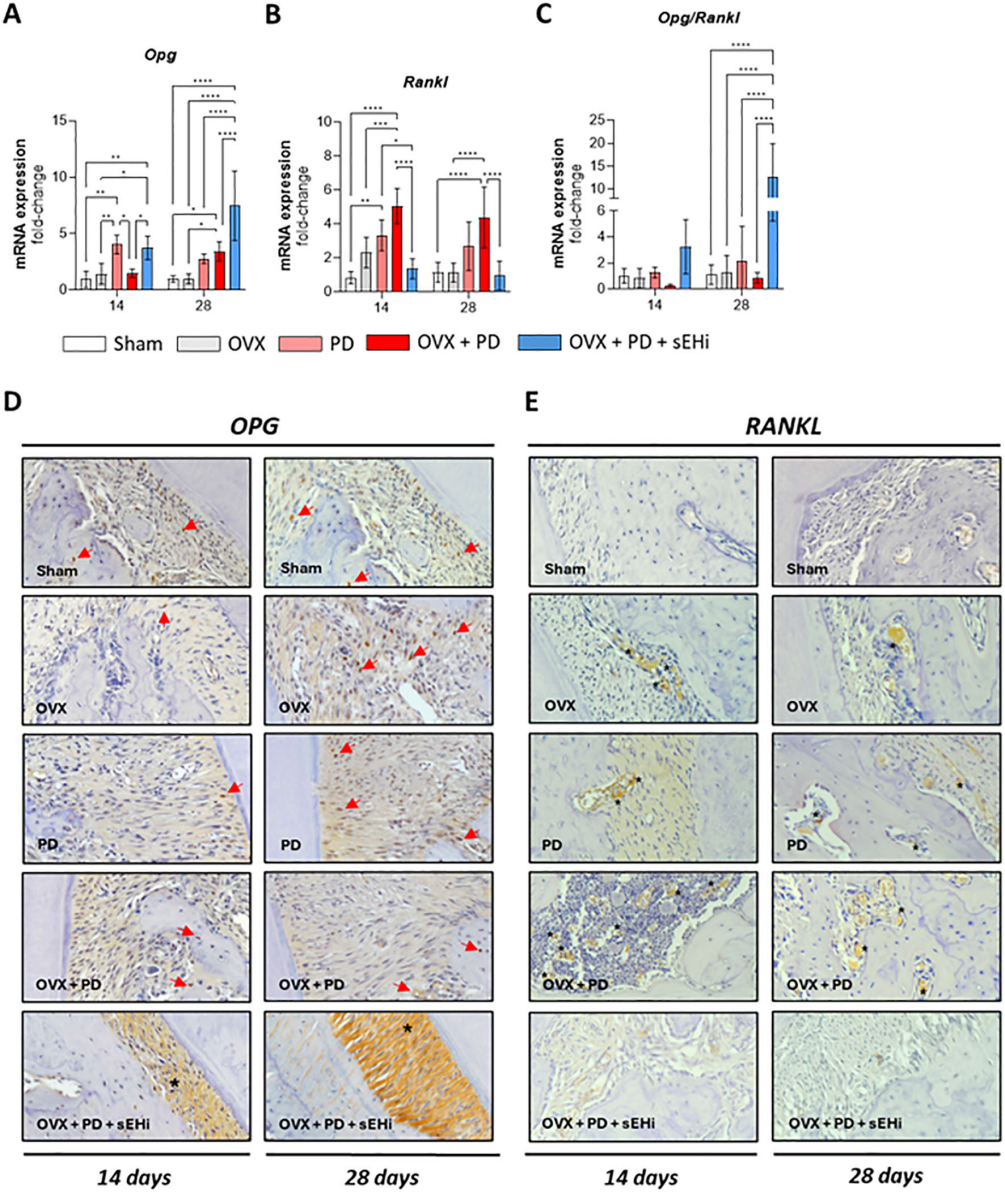
sEH inhibition upregulated OPG/RANKL ratio under estrogen-deficient conditions. mRNA expression of **(A)** Rankl, **(B)** Opg, and **(C)** Opg/Rankl ratio. Immunohistochemistry of **(D)** RANKL (The asterisk indicates positive immunostaining in the periodontal ligament. Red arrows indicate positive nuclear immunostaining in periodontal ligament cells and/or within the bone matrix, resembling osteocytes); and **(E)** OPG (The asterisk indicates positive immunostaining on leukocyte cells present in inflammatory infiltrate and/or near the alveolar bone). Representative immunohistochemistry images in each group. Magnification: x200. Data are expressed as mean ± SD; n = 5 animals per group. *p<0.05, **p < 0.01, ***p < 0.001, ****p < 0.0001.

Consistent with Trap results, Rankl expression was significantly increased in PD and OVX+PD groups at both time points (p<0.05), an effect counteracted by sEH inhibition (p<0.05; **Figure 4B**). Conversely, Opg expression was significantly elevated in OVX+PD+sEHi compared with all other groups across both time points (p<0.05; **Figure 4A**). Additionally, the Opg/Rankl ratio revealed a significant increase in the sEHi-treated group at 28 days (p<0.05), indicating a shift toward OPG dominance and reinforcing the protective effect of TPPU against alveolar bone loss (**Figure 4C**). Next, the immunohistochemical images revealed that the PD+OVX+sEHi group showed strong OPG immunostaining at 14 and 28 days, particularly within the periodontal ligament, whereas the other groups showed either absence of staining (CTL) or weak labeling (**Figure 4D**). Finally, the OVX, PD, and OVX+PD groups exhibited positive RANKL immunostaining, especially within leukocytic infiltrates, while the CTL and PD+OVX+sEHi groups exhibited low RANKL immunolabeling (**Figure 4E**).

### 3.3 sEH inhibition modulates inflammatory cytokines linked to bone loss and regulates the Th17/Treg balance

**Figure 5:**
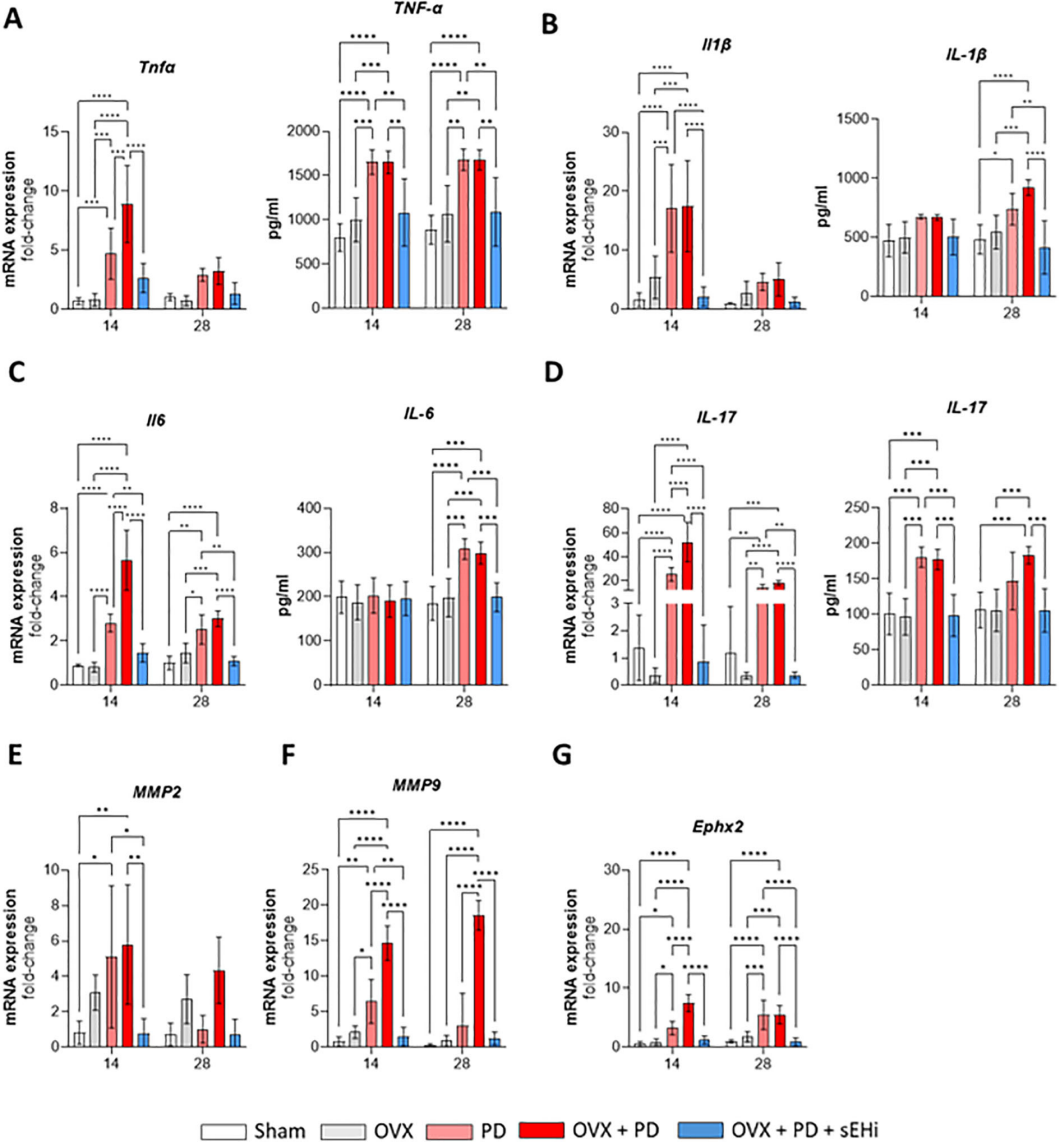
Pharmacological inhibition of sEH reduces inflammatory milieu in gingival tissue. Gene expression and protein level of **(A)** Tnfα, **(B)** Il1β, **(C)** Il6, **(D)** Il17; Gingival mRNA expression of **(E)** Mmp2, **(F)** Mmp9, **(G)** Ephx2. Data are expressed as mean ± SD; n = 5 animals per group. *p<0.05, **p < 0.01, ***p < 0.001, ****p < 0.0001.

Subsequently, we analyzed the gene expression and protein level of pro-inflammatory cytokines in gingival tissue (**Figure 5**). At 14 days post ligature-induced periodontitis, gene expression and protein level of Tnf-α (**Figure 5A**) was significantly upregulated in both PD and OVX+PD groups compared with Sham and OVX controls (p<0.05). Treatment with the sEH inhibitor attenuated this increase, bringing Tnf-α expression closer to basal levels (p<0.05) (**Figure 5A**); however, no differences were found at 28 days (p>0.05) for mRNA expression, while protein level maintained the same pattern across all groups (**Figure 5A**). Likewise, gene expression of Il-1β (**Figure 5B**) followed the same trend, with PD and OVX+PD groups showing strong induction of this cytokine at both time points (p<0.05), whereas pharmacological inhibition of sEH markedly reduced its expression (p<0.05). Nevertheless, the protein level of IL-1β exhibited statistical significance in 28 days, where PD and OVX+PD showed the highest protein levels of IL-1β and, the treatment with the sHEi abrogated it down (p<0.05; **Figure 5B**).

Regarding gene expression of Il-6 (**Figure 5C**), a robust increase was detected in PD and OVX+PD animals at 14 and 28 days (p<0.05), and once again, sEH inhibition significantly counteracted this response (p<0.05). In contrast, IL-6 protein levels showed a significant increase in PD and OVX+PD groups at the 28-day time point, being reduced by the sEH inhibition (p<0.05; **Figure 5C**), mirroring the pattern observed for IL-1β. Concerning Il-17 gene expression (**Figure 5D**), both PD and OVX+PD groups exhibited a significant increase at 14 and 28 days compared to Sham and OVX controls (p<0.05). The highest levels were observed in the OVX+PD condition, indicating an additive effect of estrogen deficiency and periodontal inflammation. Treatment with the sEH inhibitor significantly reduced Il-17 gene expression at both time points (p<0.05), with values approaching those of non-inflamed controls (Sham) at 28 days. Regarding IL-17 protein levels, a pattern consistent with its gene expression was observed. The PD and OVX+PD groups showed a significant increase at both 14 and 28 days after ligature placement, whereas treatment with the sEH inhibitor markedly reduced these levels (p<0.05; **Figure 5D**).

Next, we assessed matrix-degrading enzymes, called matrix metalloproteinase-2 and 9 (MMP). Mmp2 (**Figure 5E**) expression was elevated in PD and OVX+PD groups at both experimental time points compared to controls (p<0.05). The sEH inhibitor significantly reduced Mmp2 transcription by 14 days (p<0.05). Similarly, Mmp9 (**Figure 5F**) expression was strongly induced in PD and OVX+PD groups at both 14 and 28 days (p<0.05). Notably, inhibition of sEH was sufficient to blunt this upregulation in both time points (p<0.05), indicating a protective effect on extracellular matrix integrity.

Finally, we examined the expression of Ephx2 (**Figure 5G**), the gene encoding soluble epoxide hydrolase, the molecular target of TPPU. A significant increase in Ephx2 expression was observed in PD and OVX+PD groups at both 14 and 28 days compared with Sham and OVX (p<0.05). Importantly, treatment with the sEH inhibitor reduced its expression at both time points (p<0.05), confirming the efficacy of the pharmacological intervention. Overall, these data demonstrate that the inhibition of sEH not only suppresses inflammatory cytokines but also downregulates matrix metalloproteinases and its own enzymatic target, thereby exerting a protective role in the context of experimental periodontitis and estrogen deficiency.

**Figure 6:**
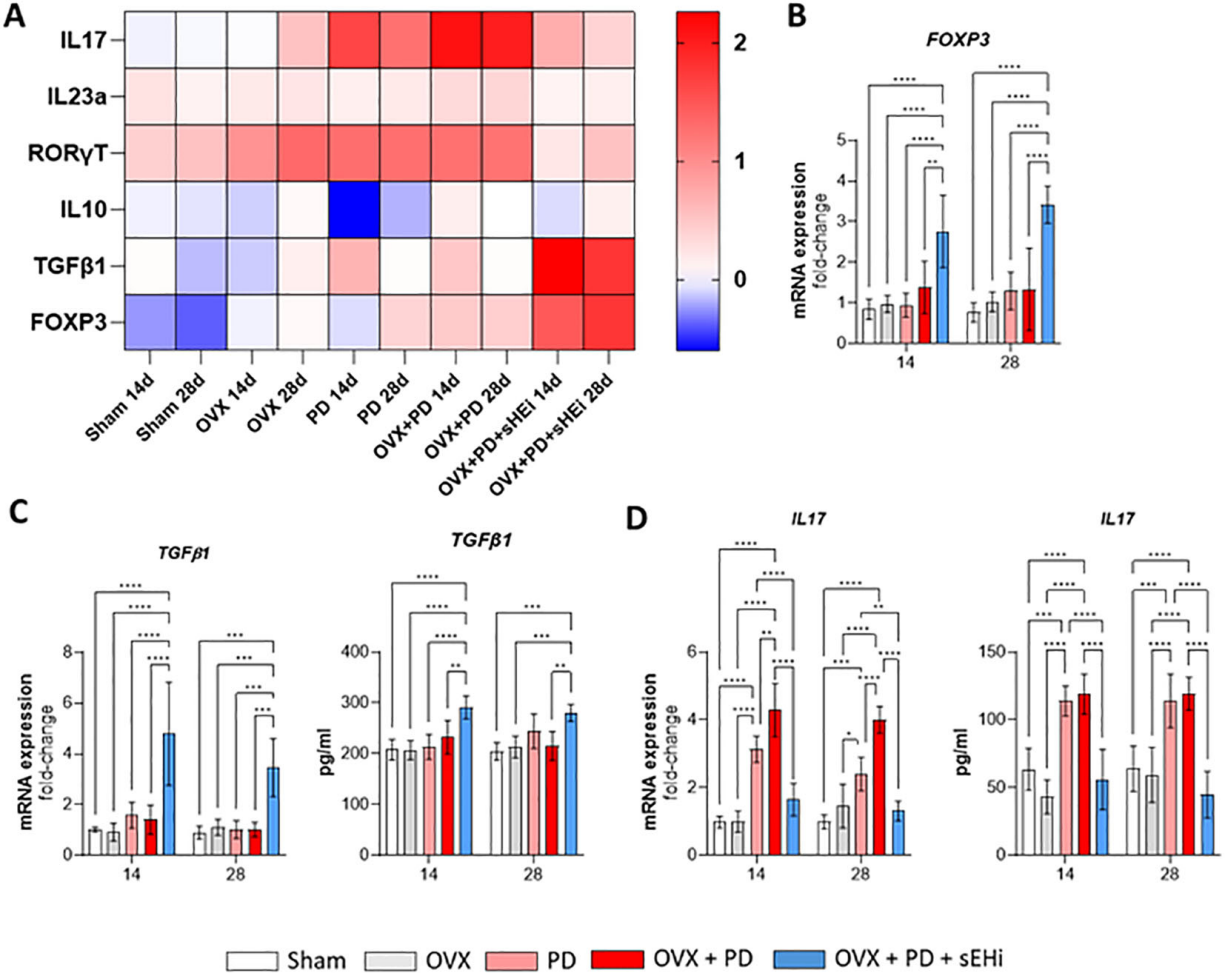
sEH inhibition restores the Treg/Th17 balance in cervical lymph nodes under estrogen-deficient conditions. **(A)** Heatmap plotted in log2 fold change of marker mRNA expression in cervical lymph nodes. mRNA expression of **(B)** Foxp3; Gene expression and protein level of **(C)** Tgfβ1, **(D)** Il17 in cervical lymph nodes. Data are expressed as mean ± SD; n = 5 animals per group. *p<0.05, **p < 0.01, ***p < 0.001, ****p < 0.0001.

To further investigate the regulation of the Treg/Th17 axis, we analyzed the transcriptional profile of cervical lymph nodes. The overall transcriptional profile, illustrated in the heatmap (**Figure 6A**), revealed distinct clustering patterns among experimental groups, indicating that estrogen deficiency and pharmacological intervention modulated immune-related pathways in divergent directions. Foxp3 mRNA expression was significantly upregulated at both time points following sEH inhibition (p < 0.05) (**Figure 6B**). Similarly, Tgfβ gene expression and protein level was also significantly increased in the sEH-inhibited group compared with the other groups (p < 0.05) (**Figure 6C**). Likewise, IL-17 gene expression and protein level was significantly elevated in the PD and OVX+PD groups compared with Sham and OVX controls (p < 0.05), whereas sEH inhibition markedly suppressed this increase (p < 0.05) (**Figure 6D**). Collectively, these findings demonstrate that sEH inhibition reestablishes, at least in part, the disrupted Treg/Th17 balance induced by estrogen deficiency, shifting the local immune milieu toward a more regulated state.

## 4 Discussion

The present study demonstrates, for the first time, that pharmacological inhibition of soluble epoxide hydrolase (sEH) with TPPU significantly attenuates alveolar bone loss in an estrogen-deficient rat model of experimental periodontitis. These findings provide novel evidence that targeting the sEH pathway represents a promising therapeutic approach to counteract the synergistic effects of menopause-related bone loss and periodontal inflammation.

Consistent with previous reports, ovariectomy induced systemic bone alterations characterized by reduced trabecular bone volume and increased porosity, validating the osteoporotic-like condition in our model (3, 4). Estrogen deficiency is known to disrupt bone homeostasis by enhancing osteoclastogenesis and impairing bone formation, mechanisms that extend to the alveolar bone and exacerbate periodontal destruction (5). Our results corroborate this evidence, as OVX animals exhibited increased alveolar bone loss, particularly when combined with periodontitis, supporting the concept that postmenopausal status is a significant risk factor for periodontal disease progression.

Building on this line of investigation, our research group has conducted a series of preclinical studies to evaluate the impact of specific osteoporosis treatments on periodontal bone preservation. In a rat model of ligature-induced periodontitis, we demonstrated that strontium ranelate—a medication with both antiresorptive and osteoanabolic properties—was effective in reducing alveolar bone loss under both estrogen-sufficient and estrogen-deficient conditions, decreasing bone resorption markers such as RANKL, alongside increased expression of bone formation markers like osteocalcin and osteopontin. These findings suggest that strontium ranelate modulates the bone remodeling process within the periodontium, even in the context of estrogen deficiency (17). In addition, we investigated the effects of lithium chloride, a GSK-3β inhibitor with established osteogenic potential, on alveolar bone loss in the same experimental model. Immunohistochemical analyses further revealed enhanced expression of osteogenic markers and modulation of OPG/RANKL balance, indicating a protective effect against periodontal bone destruction (18). These results underscore the potential dual benefit of certain osteoporosis medications for both systemic and oral bone preservation, and support the rationale for future translational research evaluating their role in periodontal therapy, especially for postmenopausal or osteoporotic patients at increased risk for periodontitis progression.

The protective effect of sEH inhibition observed in this study appears to involve multiple mechanisms, including modulation of osteoclast differentiation and activity. Histological and molecular analyses demonstrated a reduction in multinucleated TRAP-positive cells and downregulation of RANKL, accompanied by an upregulation of OPG and a higher OPG/RANKL ratio, suggesting a shift toward bone preservation. These findings are in line with previous evidence indicating that EETs and other epoxy fatty acids, stabilized by sEH inhibition, exert anti-inflammatory and pro-resolving actions that indirectly regulate osteoclastogenesis (11, 13). In addition, previous evidence shows that the anti-inflammatory and pro-resolutive actions of sEH inhibition appear to be closely linked to its capacity to modulate endothelial–immune interactions and osteoclast function (26). By upregulating endomucin (EMCN), a salivary glycoprotein, TPPU reduces endothelial adhesion (reducing neutrophil migration) and downstream osteoclast activation, a mechanism that aligns with the emerging role of vascular regulation in periodontal breakdown (26). These data support the bone-preserving and immunomodulatory effects observed in the present work.

Mechanistically, accumulating evidence indicates that the EET/sEH axis converges on key transcriptional regulators governing T-cell polarization and bone metabolism. In innate immune cells, EETs activate the peroxisome proliferator–activated receptors gamma (PPARγ) and heme oxygenase-1, suppressing NF-κB activation and reducing the production of IL-6 and IL-1β, cytokines that strongly promote Th17 differentiation (27, 28). In line with these findings, recent findings shows that pharmacological inhibition or genetic deletion of sEH markedly attenuates NF-κB–driven inflammatory signaling and its downstream mediators (15, 29–31). In addition, sEH inhibition has been shown to downregulated STAT3 phosphorylation in a neuroinflammation model in astrocyte (32), a transcription factor recognized as central to the reciprocal differentiation of Th17 and Treg cells (33). Beyond immune modulation, recent evidence demonstrates that sEH inhibition enhances osteoblast differentiation and bone-forming capacity, suggesting that EET stabilization may simultaneously influence skeletal and immunological pathways (34).

Moreover, sEH inhibition effectively suppressed the expression of key pro-inflammatory cytokines (IL-1β, IL-6, TNF-α) and matrix metalloproteinases (MMP2, MMP9), both of which play critical roles in periodontal tissue breakdown (35, 36). Notably, the inhibitor also modulated the Th17/Treg axis by reducing IL-17 expression and enhancing Foxp3 and TGF-β expression in cervical lymph nodes, suggesting a restoration of immune homeostasis. These findings are particularly relevant given the well-established role of Th17 cells in osteoclast activation and bone loss in periodontitis (12, 37).

When compared to conventional antiresorptive therapies, such as bisphosphonates and denosumab, which effectively reduce skeletal fractures but carry a risk of medication-related osteonecrosis of the jaw (6, 8), sEH inhibitors offer a distinct advantage by promoting resolution of inflammation without impairing physiological bone remodeling. This mechanism may provide a safer alternative for managing bone loss associated with systemic osteoporosis and periodontitis.

In contrast to conventional anti-resorptive agents such as bisphosphonates and denosumab—which effectively reduce bone resorption but may impair bone turnover and increase the risk of MRONJ (38, 39)—sEH inhibition modulates inflammation and the incidence of osteoclast-like cells without directly suppressing remodeling. This distinct mechanism may offer safety advantages; however, no studies have directly compared TPPU with anti-resorptive drugs, limiting direct therapeutic comparisons and underscoring the need for future research.

Taken together, these findings highlight the therapeutic potential of sEH inhibitors as modulators of both inflammatory and bone-resorptive pathways in conditions of estrogen deficiency. Nevertheless, our study has limitations. The animal model used does not fully reproduce the complexity of human menopause and periodontitis. In addition, TPPU was administered before ligature placement, representing a preventive rather than a therapeutic model. Future studies should evaluate the efficacy of sEH inhibition once periodontal disease is established, as well as focus on dose optimization, long-term safety, and potential synergistic effects with current osteoporosis therapies to better define the translational relevance of these findings.

## 5 Conclusion

In summary, our findings demonstrate that pharmacological inhibition of soluble epoxide hydrolase (sEH) with TPPU effectively attenuates alveolar bone loss in an estrogen-deficient model of experimental periodontitis. This protective effect involves suppression of pro-inflammatory cytokines and matrix metalloproteinases, modulation of the OPG/RANKL axis, and restoration of the Th17/Treg balance, ultimately reducing osteoclast activity and preserving periodontal architecture. By targeting both inflammatory and bone-resorptive pathways without impairing physiological bone remodeling, sEH inhibition emerges as a promising therapeutic strategy for managing bone loss associated with postmenopausal osteoporosis and periodontitis. Further translational studies are warranted to confirm these findings, optimize dosing regimens, and evaluate potential synergistic effects with current osteoporosis and periodontal therapies.

## Data Availability

The original contributions presented in the study are included in the article/supplementary material. Further inquiries can be directed to the corresponding author.
